# Role of Overload and Psychoemotional Variables on Health-Related Quality of Life in Informal Caregivers of People with Alzheimer’s Disease

**DOI:** 10.3390/jcm13206188

**Published:** 2024-10-17

**Authors:** Patricia Ferrero-Sereno, Patricia Palomo-López, María Mendoza-Muñoz, Jorge Carlos-Vivas, Javier Urbano-Mairena, Laura Muñoz-Bermejo

**Affiliations:** 1 Social Impact and Innovation in Health (InHEALTH), University Centre of Mérida, University of Extremadura, 06800 Merida, Spain; pferrser@uax.es (P.F.-S.); jurbmai@unex.es (J.U.-M.); lauramunoz@unex.es (L.M.-B.); 2 University Center of Plasencia, University of Extremadura, 06006 Badajoz, Spain; 3 Research Group on Physical and Health Literacy and Health-Related Quality of Life (PHYQOL), Faculty of Sport Sciences, University of Extremadura, 10003 Caceres, Spain; 4 Physical Activity for Education, Performance and Health (PAEPH) Research Group, Faculty of Sport Sciences, University of Extremadura, 10003 Cáceres, Spain; jorgecv@unex.es

**Keywords:** Alzheimer’s disease, caregivers, overload, mental health, quality of life

## Abstract

**Background:** Carers of people with Alzheimer’s disease often have a high degree of commitment and dedication which may also compromise physical and emotional, leisure, and occupational self-care. This study aimed to explore health-related quality of life (HRQoL) and psychoemotional variables in caregivers with and without caregiver overload and its relationship. **Methods:** A single-measure cross-sectional correlational study was carried out involving 59 informal caregivers of people with Alzheimer’s disease with a mean age of 59.30 (±10.58). The participants completed the adult HRQoL questionnaires (EQ-5D-3L), Zarit Burden Inventory test, General Happiness Questionnaire, Satisfaction with Life Scale, Rosenberg self-esteem scale, Occupational Balance Questionnaire (OBQ-E), International Fitness Scale (IFIS), Family Apgar scale, and Duke-UNC-11 Functional Social Support Questionnaire. **Results:** A significantly higher level of HRQoL (*p* = 0.029) in subjective happiness (*p* = 0.018), perceived social support (*p* = 0.046), avoidance (*p* = 0.034), occupational balance (*p* = 0.002), life satisfaction (*p* = 0.037), and self-perceived physical fitness (*p* = 0.021) was found in caregivers without perceived overload. Also, HRQoL was directly associated with self-perceived physical fitness (β = 0.534; *p* < 0.001) and occupational balance (β = 0.375; *p* < 0.001) and self-esteem (β = 0.249; *p* < 0.016). **Conclusions:** Caregivers who do not perceive overload have better levels of HRQoL and psychoemotional variables, establishing a relationship between HRQoL with self-perceived physical fitness, occupational balance, and self-esteem.

## 1. Introduction

Alzheimer’s disease (AD) is considered the leading cause of dementia worldwide. The disease presents urgent challenges in terms of clinical care, caregiving, and family and caregivers [1].

AD presents insidiously and has a progressive course causing a gradual loss of the ability to perform routine and self-care tasks, increasing the need for supervision and care by others [2]. Care for patients with AD is carried out primarily by family members, thus becoming family caregivers. This care is mostly provided by spouses and children at home [3,4]. These carers usually have a high degree of commitment to the care of their family member. The care provided is characterized by affection and care without time limits. They often have no specific training and do not receive financial remuneration for their work [5]. In addition, the caregiver may have to undertake activities that may not motivate them or for which they do not feel prepared [6]. The need for attention and care can be continuous and sometimes unpredictable, so the care required by the sick person sometimes exceeds the capabilities of the caregiver, who turns caregiving into a chronic stressful event that generates overload [7]. In this regard, due to the high demand for care and an undetermined schedule, caregivers are often subject to a physical and emotional burden related to care [8].

By spending too much time caring for their family members with AD, caregivers often exhibit a low level of self-care associated with signs of depression, anxiety, and occupational stress, affecting their physical and mental health [9]. Therefore, AD is a dementia that conditions the patient’s quality of life (QoL) and at the same time has an impact on the caregiver’s quality of life [10]. Studies in Western populations indicate that good physical health and good mental health are associated with better caregiver QoL. Physical health enables caregivers to have the energy and stamina to perform daily caregiving tasks. Good mental health helps caregivers manage the stress and anxiety that can arise from the responsibility of caring for others. In addition, adequate rest, sleep, and time allow the body to recover from physical fatigue and improve irritability. This allows them to maintain a more positive and resilient attitude in the face of challenges. [11,12].

Older caregivers, moreover, are aware that decreased muscle mass may condition the ability to perform routine caregiving. Therefore, physical fitness may decrease the caregiver’s efforts in strength tasks and improve the ability to perform caregiving tasks [13]. In this sense, caregivers exhibit a lower HRQoL than non-carers in all QoL dimensions, except in the physical function dimension, referring to the health limitation to perform the physical activities of daily living [14,15].

On the other hand, in terms of mental health, it is important to note that psychoemotional variables such as happiness, life satisfaction, avoidance, overload, and perceived social and family support are important factors that could influence the caregiver’s mental health status and, ultimately, quality of life. In this sense, happier caregivers who are more satisfied with life have better physical health, use less avoidant coping, experience less overload, have more social support (confidential and affective), have better mental health and quality of life than less happy caregivers, and have better mental health and quality of life than less happy caregivers [16]. In addition, social support is confirmed as a mediating variable between overload, risk of anxiety and depression, and quality of life [17].

The area of care presents the interrelationship and interdependence between the caregiver’s level of physical function, social systems, and the activities or care to be performed, characterizing a balance between the needs and skills of the caregiver and the demands of the caregiver [18]. Situations of overload can have negative consequences on the occupational performance of informal carers, defined as the person’s ability to carry out their occupations (basic and instrumental activities of daily living, studies/work, leisure, social life, and disconnection and rest) [19].

Overburdened caregivers report poorer mental health [20], poorer occupational balance, and poorer subjective health and well-being [21], all variables that can affect the caregiver’s quality of life as a whole or individually. Therefore, it is necessary to study the influence of these variables on the caregiver’s quality of life. Therefore, the main objectives were (1) to explore the health-related quality of life and psychoemotional variables (happiness, life satisfaction, self-esteem, family functioning, occupational balance, perceived physical condition, and avoidance) in the informal caregivers of people with Alzheimer’s disease; (2) to analyze the differences between caregivers with and without caregiver strain; and (3) to analyze the influence of psychoemotional variables on caregivers’ quality of life.

## 2. Materials and Methods

### 2.1. Design

A single measure, cross-sectional, descriptive, and correlational study was conducted to examine the health-related quality of life and psychoemotional variables in informal caregivers of people with Alzheimer’s disease; to explore the differences between caregivers with and without caregiver strain; and to analyze the influence of psychoemotional variables on caregivers’ quality of life.

### 2.2. Sample Size Calculation

A total of 43 participants were needed to reach 80% power, accepting an effect size f^2^ of 0.15 and an alpha risk of 0.05.

### 2.3. Participants

The sample consisted of a total of 59 informal caregivers of people with Alzheimer’s disease.

The following inclusion criteria were established for participation in the study: (1) being an informal caregiver of a person with Alzheimer’s; (2) performing caregiving tasks for more than 20 h a week and having been performing these tasks for at least three months and having the willingness to continue caring for their relative for one year; (3) not suffering from pathologies that constrain physical activity or special adaptations, such as coronary pathologies, thrombosis, and symptoms associated with COVID-19, among others; (4) not have participated in physical activity programs during the three months prior to the start of this program; (5) not have participated in psychoeducational or cognitive–behavioral sessions in the three months prior to this intervention; (6) have signed the informed consent form for the study and given it to a member of the research team prior to the start of the intervention.

Caregivers who received any type of financial compensation for their service were excluded.

### 2.4. Ethics

The research received approval from the Bioethics and Biosafety Committee of the University of Extremadura (approval number: 145/2024) in accordance with the revisions made to the Declaration of Helsinki by the 64th General Assembly of the World Medical Association (Fortaleza, Brazil, 2013) and in compliance with Law 14/2007 on Biomedical Research.

### 2.5. Procedures and Measures

Data were collected from each caregiver personally and by means of a standardized questionnaire self-administered in associations of the relatives of people with AD. Both the interview and the measurements were previously arranged by the research team and were carried out in July 2024.

Quality of life: EQ-5D-3L, this questionnaire assesses the health status of participants. It consists of three parts: (1) a descriptive system with five levels of severity, assessing several dimensions of health (mobility, self-care, activities of daily living, pain/discomfort, and anxiety/depression) with a scale from one (no problems) to five (problems/external impossibility); (2) a visual analog scale; and (3) a social value index generated from the health states obtained in the first level [22]. This instrument proved to be valid and reliable in both young [23] and adult populations [24].

Caregiver strain: The abbreviated Zarit caregiver burden scale was used to assess caregiver strain. This 7-item questionnaire is in Likert scale format, using a scale from one (never) to five (almost always). The sum of all the items reflects the degree of caregiver strain. Values less than or equal to 16 points were considered the absence of caregiver strain and caregivers with caregiver strain. The scale showed a Cronbach’s alpha of 0.84 in the Spanish population [25,26]. The abbreviated Zarit demonstrated its validity and reliability in caregivers with different conditions [27].

Self-esteem: The Rosenberg self-esteem scale is a widely used instrument that quantifies individuals’ self-satisfaction by assessing the alignment between their ideal self-image and their perceived reality. This scale has been adapted into Spanish with robust psychometric properties, demonstrating high internal consistency (Cronbach’s alpha = 0.87). Moreover, it proved to be valid and reliable in the Spanish population [28]. The instrument showed a good fit index (Chi square = 217.20, *p* < 0.05; CFI = 0.965; GFI = 0.980; RMSEA = 0.070 [90% confidence interval of RMSEA, 0.022–0.087]) in Spanish adults [29].

Happiness: The Subjective Happiness Scale (SHS) serves as a comprehensive tool for evaluating subjective happiness, which assesses a general category of well-being as a global psychological phenomenon considering the definition of happiness from the perspective of the respondent. This instrument is structured with 4 Likert-type response items, designed to capture varying facets of happiness based on individual perspectives. The scores are aggregated by summing responses and dividing them by the total number of items, providing a quantitative measure of overall happiness. The questionnaire has demonstrated robust internal consistency across diverse samples, spanning different age groups, occupations, languages, and cultural backgrounds [30]. The internal consistency coefficient for the Spanish version of the SHS was 0.81 for the total sample (Cronbach’s alpha = 0.80 and 0.82 for men and women, respectively). Also, adequate goodness-of-fit indices were found (CFI = 0.99; GFI = 0.99; RMSEA = 0.06; SRMSR = 0.02) of the Spanish SHS [31]. The Spanish version of the SHS has proven to be valid and reliable for the adult population [32,33].

Satisfaction with Life: The Satisfaction with Life Scale (SWLS) is a concise tool designed to evaluate individuals’ overall life satisfaction. Originally comprising 5 items scored on a 1 to 7 scale and adapted in the Spanish version to a 1 to 5 scale, respondents indicate their agreement from “totally disagree” to “totally agree”. This version has good psychometric properties. The reliability index calculated for the Cronbach’s alpha scale indicates that the scale has a very good internal consistency (Cronbach’s alpha = 0.84) [34]. The internal consistency of the scale in Spanish adults was Cronbach’s alpha= 0.88. The unifactorial structure of the SWLS (Chi square = 38.46, *p* < 0.05; CFI = 0.994; TLI = 0.988; RMSEA = 0.047) was confirmed in Spanish adults [35]. It has been shown to be a valid and reliable instrument for measuring overall life satisfaction in the general population [36,37].

Cognitive–behavioral aspects: The Spanish version of the Cognitive–Behavioral Avoidance Scale (CBAS) was used. This scale comprises 31 items that assess different strategies of avoidance coping, categorized into four distinct factors: behavioral/social, behavioral/non-social, cognitive/social, and cognitive/non-social. The participants rated their agreement on a five-point Likert scale, ranging from “Not so true for me” to “Extremely true for me”, providing a comprehensive evaluation of avoidance tendencies across multiple domains. A high score indicates more avoidance. The CBAS demonstrated robust internal consistency with a Cronbach’s alpha coefficient of 0.8. Moreover, construct validity was confirmed in Spanish adults (Chi square = 660.82, *p* < 0.05; CFI = 0.92; RMSEA = 0.040) [38]. The CBAS proved its validity in the general population, clinical patients, and adult learners [39].

Occupational Balance: The Occupational Balance Questionnaire (OBQ-E) allows the assessment of participants’ satisfaction with their occupations, using 13 items, answered on a Likert scale from zero “strongly disagree” to five “strongly agree” [40]. The OBQ showed good internal consistency (α-Cronbach = 0.87), intraclass reliability (ICC = 0.87), and test–retest reliability (rho = 0.83). It proved to be a valid and reliable measurement instrument in Spanish adults [41].

Perceived social support: the Duke-UNC-11 Functional Social Support Questionnaire was used to determine the perceived social support of older caregivers. This instrument features 11 items designed on a Likert response scale, ranging from 1 (“much less than I want”) to 5 (“as much as I want”), thereby encompassing various dimensions of social support. Scores derived from this questionnaire range from 11 to 55 points. The Spanish adaptation of the Duke-UNC-11 questionnaire utilizes a threshold at the 15th percentile, with a corresponding score of less than 32 denoting lower perceived social support, while a score of 32 or above indicates a standard level of support. The internal consistency within the Spanish population was notably high, measuring at 0.90, affirming the reliability of this tool in Spanish-speaking communities. Moreover, the intraclass correlation coefficients for the 11 Duke-UNC-11 items were above 0.50 in Spanish adults [42]. The questionnaire has proven to be reliable and valid in caregivers in Spain [43].

Family Functionality: Family functionality was evaluated using the Family Apgar scale, a tool designed to gauge the respondents’ satisfaction with their family relationships and overall functioning. Comprising five Likert-type items, where responses range from 0 (“practically never”) to 2 (“practically always”), this scale provides a structured assessment of familial dynamics based on perceived satisfaction levels. The scale categorizes family functionality into three distinct levels: functional family (7–10 points), slightly dysfunctional family (4–6 points), and severely dysfunctional family (0–3 points), offering clear thresholds for interpreting varying degrees of family dysfunctionality. It has high internal consistency, as indicated by a Cronbach’s alpha of 0.84 [44]. Validity was demonstrated with excellent goodness-of-fit indicators (Chi square = 7.90; *p* = 0.162; RMSEA = 0.04; TLI = 0.99; CFI = 0.99) in the adult population [45]. The Family APGAR is a reliable and appropriate instrument to be applied in older people [46,47].

Self-perception of physical fitness: The International Fitness Scale (IFIS) [48] was used to assess self-perceived physical fitness. This instrument consists of 5 items in the form of a 5-Likert scale (general fitness, muscular strength, cardiorespiratory fitness, speed–agility, and flexibility). The response options are “very poor”, “poor”, “fair”, “good”, and “very good”. A higher score means a better perception of physical fitness. The Spanish version proved to be valid and reliable [49] in older adults and in Spanish young adults [50].

### 2.6. Statistical Analysis

All the information collected during the intervention was coded in a database designed specifically for the participating caregivers. The data are presented as mean and standard deviation (continuous variables) and absolute and relative frequencies (ordinal variables). The Kolmogorov–Smirnov test was applied to check that the sample distribution data for every variable fit a normal distribution. The results showed that all the variables did not fit a normal distribution. Thus, between-group differences were analyzed using the Mann–Whitney U-test for continuous variables and the χ^2^ test for categorical variables. The significant level was set at a *p*-value of 0.05 for all the tests.

Independent Student t-tests were used to establish the differences between groups of the values of the variables with normal distribution and the Mann–Whitney U tests were used to know the differences between groups of the variables with non-normal distribution. Significant differences were considered for *p* ≤ 0.05. Specific regressions were used to study the effects of the predictor variables (psychoemotional variables) on the caregivers’ quality of life. Bilateral *p*-values ≤ 0.05 were considered statistically significant. The overall predictive power was evaluated by adjusted R2. Statistical analysis was conducted using the Statistical Package for the Social Sciences (SPSS, Version 25, IBM SPSS, Armonk, NY, USA) software.

## 3. Results

The sociodemographic, economic, and anthropometric characteristics of the participants and their comparison according to the perception or not of caregiving overload are shown in Table 1. It can be seen that these comparisons revealed no statistical differences between the two groups of caregivers.

Table 2 shows the psychoemotional variables and the differences between the groups of caregivers with and without caregiving overload. Comparisons between the caregivers with and without perceived caregiving burden revealed a significantly higher level of quality of life in the caregivers without perceived caregiving burden (*p* = 0.029). In addition, significant differences were found in subjective happiness (*p* = 0.018), perceived social support (*p* = 0.046), avoidance (*p* = 0.034), occupational balance (*p* = 0.002), life satisfaction (*p* = 0.037), and perceived physical condition (*p* = 0.21).

Table 3 shows the stepwise regression model results obtained for two different models. The first model shows that HRQoL can be explained by perceived physical fitness (IFIS). Specifically, QoL is directly associated with IFIS (β = 0.688; *p* < 0.001). The second model directly relates QoL to perceived physical fitness (IFIS) (β = 0.580; *p* < 0.001) and occupational balance (OBQ) (β = 0.395; *p* < 0.006). And finally, the third model directly relates QoL to self-perceived physical fitness (β = 0.580; *p* < 0.001), occupational balance (β = 0.375; *p* = 0.001), and self-esteem (β = 0.249; *p* = 0.016).

## 4. Discussion

This study investigated the psychoemotional variables perceived by the caregivers of people with Alzheimer’s disease and the differences between the groups with and without perceived caregiving burden. It also explored the effect of these variables on the caregivers’ quality of life. The main finding was that the caregivers with better perceived physical fitness and good occupational balance would predict a better quality of life. A positive association was found between the quality of life and IFIS and OBQ.

Although the overburdened caregivers perceive a lower quality of life than the caregivers who do not perceive overburden, this has not been a predictor of the caregivers’ level of quality of life. However, in other studies, the level of AD burden extends to caregivers in terms of health-related quality of life (HRQoL) [51,52]. This may be conditioned by the fact that caring for a person with AD requires a high level of tasks on the part of the caregiver and a long-term commitment to their care. It is a progressive disease with the deterioration of the patient’s functional capacity, cognitive abilities, and behavioral and personality disorders. In addition, as time goes by, the need for care increases, thus increasing the level of dedication of the caregiver. [53]. Whether the outcome of this performance is satisfactory or not, in influencing the quality of life, depends on the interaction between a variety of elements that are in a continuous relationship: caregiver characteristics (values, beliefs, and spirituality; bodily functions and structures), performance skills, performance patterns (habits, routines, rituals, and roles), and contexts and environments [54]. Intrinsic motivations such as love, responsibility, or empathy are critical to caregiver well-being, as they can help mitigate the emotional burden that often comes with the caregiving role. Extrinsic motivations can also influence how caregivers approach their role and gain recognition from the community. Among the main reasons for performing caregiving for patients with dementia are moral obligation, personal dignity and satisfaction, gratitude to the ill person, social pressure, and not having the financial means for institutionalization [55]. Carers by choice reported better QoL, so the decision to become a carer could be central to the carer’s adjustment to their relative’s illness. In this sense, it could be thought that people who are carers by choice would experience positive feelings as a result of the caregiving role. This could attenuate the impact of caregiver stress, promoting caregivers’ QoL [56].

The impact of different factors on caregivers’ quality of life has been studied in different research. The caregivers who showed better QoL were younger caregivers belonging to a normofunctional family [57], those who received help in caregiving tasks and caregivers with chronic diseases [51,52], those who cared for relatives in the early stages of AD [58], and caregivers whose relative did not have severe behavioral problems [59].

According to our study, the caregivers without caregiver strain are more satisfied with life, have normal social support, higher values of subjective happiness, better occupational balance, less subjective discomfort in situations of social interaction, better perception of their physical condition, and finally, better quality of life. In other studies, the degree of caregiver strain was conditioned by the caregiver’s gender, lack of free time, number of hours of caregiving, number of years of caregiving, caregiver self-esteem [60], perceived social support [61], the neuropsychiatric profile of the patient [62], and progression of the patient’s disease [60].

The results of the Rosenberg self-esteem scale, as well as the results of the IFIS, have not obtained significant differences between the overburdened and non-overburdened caregivers. It is possible that both groups of caregivers have an awareness of the severity of the disease and feelings of comparison with other realities [63].

In our study, occupational balance contributed to improving the caregivers’ quality of life. In this sense, it is worth mentioning that occupational imbalance, related to the inadequate temporal distribution of occupations, in this case, dedicating too much time to caregiving at the expense of other areas, can generate different health problems [19]. In this sense, the caregivers who reported a better QoL were those who devoted fewer hours to caregiving and received help with caregiving tasks, thus relieving the caregiver’s burden [57]. For older adults, achieving professional balance is critical to maintaining good physical and mental health. When there is a proper balance between caregiving and personal life, stress and anxiety are reduced, which in turn reduces the risk of stress-related illnesses, such as heart problems or mental health disorders. Therefore, occupational balance may have a protective effect on health and a preventive effect on disease [64].

Carers’ perceived physical condition was also a modulator of QoL. Carers who perceived a good physical condition also identified a better QoL. It should be borne in mind that the average age of the caregivers assessed was around 60 years. At this age, decreasing muscle mass can become a significant challenge. This loss can affect strength and endurance, which in turn can make it difficult to perform everyday tasks, including caring for oneself and others. Preventing the loss of muscle mass is essential to maintaining independence and quality of life [65]. Carers are also aware of the need to maintain physical fitness in order to maintain their ability to care [13]. For this reason, the caregivers who perceive themselves to be in better physical condition consider themselves more prepared to cope with caregiving tasks and this could result in a better QoL. In addition, the caregivers of patients with AD present a lower HRQoL in all areas, except in the area of functional and physical capacity where the caregivers present higher scores [66]. 

This study has some limitations. Firstly, the sample size was relatively small and included mainly caregivers over 60 years of age. The sample was not selected using population sampling techniques; participants from associations of the family members of people with AD were sampled. However, the profile of the caregivers is similar to the profiles described in other studies previously conducted in Spain [62]. Second, the sample was taken from a specific geographic location, so the results may not be representative of other populations or contexts. Third, the cross-sectional methodology only allows us to observe relationships at a specific moment in time, which makes it difficult to establish causal relationships. Finally, self-reported measures were used, which may lead to response bias and affect the validity of the data. Overall, these results suggest that a holistic approach to caregiver needs, with particular attention to caregiver mental health and mood, may be helpful in addressing caregiver burden. The positive relationship between perceived physical fitness and quality of life suggests that the caregivers who maintain a good level of physical fitness tend to experience a better quality of life. This may be due to better physical fitness providing greater ability to cope with the physical demands of the caregiving role, such as mobilizing the dependent person, which reduces the risk of burnout. Physical activity is linked to better mental health, as it reduces stress, improves mood, and can alleviate the symptoms of anxiety and depression, common problems in caregivers.

On the other hand, they also suggest the need to analyze the tasks and occupations of the caregiver and their physical state, since both variables can determine the quality of life. The association between occupational balance and quality of life underscores the importance of preventing burnout, as the caregivers who achieve an adequate balance between their responsibilities and their own personal needs experience less emotional and physical exhaustion. The caregivers who devote time to their relationships and social networks may have a better emotional support system, which enhances their resilience to the demands of caregiving.

In summary, these implications highlight the need to comprehensively support caregivers, not only in their physical capacity, but also in their ability to maintain a balance in their different responsibilities, which, in turn, translates into a significant improvement in their quality of life.

## 5. Conclusions

The present study showed that caregivers had a better HRQoL when they did not perceive strain. In addition, the caregivers who do not experience strain have a better level of subjective happiness, perceived social support, avoidance, occupational balance, life satisfaction, and perceived physical fitness. In addition, caregivers’ quality of life is related to perceived physical fitness and occupational balance. Based on these findings, future research could focus on developing and implementing interventions designed to enhance perceived physical fitness and promote occupational balance among caregivers. Such interventions could potentially lead to significant improvements in their overall health-related quality of life.

## Figures and Tables

**Table 1:** Sociodemographic data for the total sample and segmented by the overburdened and non-overburdened participants.

	Total			No Overload			Overload			
	N	%		N	%		N	%		
**Relationship**										
Son/Daughter	37	62.7		13	61.9		24	63.2		
Partner	21	35.6		8	38.1		13	34.2		
Brother/Sister	1	1.7								
**Economic sufficiency**										
Yes	32	54.2		11	52.4		21	55.3		
No	27	45.8		10	47.6		17	44.7		
**Level of education**										
Reading and writing	5	8.5		0	0		5	13.2		
Primary education	9	15.3		5	23.8		4	10.5		
Secondary education	13	22.0		6	28.6		7	18.4		
Baccalaureate	16	27.1		6	28.6		10	26.3		
Higher education	16	27.1		4	19.0		12	31.6		
**Marital status**										
Single	11	18.6		7	33.3		4	10.5		
Married	42	71.2		12	57.1		30	78.9		
Divorced	5	8.5		2	9.5		3	7.9		
Widowed	1	1.7		0	0		0	0		
	**N**	**Mean**	**SD**	**n**	**Mean**	**DT**		**Mean**	**SD**	***p***
Age (years)	59	59.30	10.58	21	59.71	10.27	38	59.08	10.89	0.827
Years of care	56	14.05	10.130	19	15.89	11.14	37	13.11	9.60	0.335
BMI (kg/m^2^)	56	27.31	4.21	20	26.58	3.50	36	27.72	4.56	0.336
WHR (cm)	51	0.85	0.083	20	0.87	0.097	31	0.84	0.072	0.189

BMI: body mass index; WHR: waist–hip ratio.

**Table 2:** Psychoemotional variables of the total sample and segmented by the overloaded and non-overloaded participants.

	Total			No Overload			Overload			
	n	Mean	SD	n	Mean	SD	n	Mean	SD	*p*
EuroQoL-5D-3l	59	0.83	0.19	21	0.90	0.08	38	0.79	0.21	0.029
Zarit Burden Inventory test	59	19.08	6.38	21	12.61	2.55	38	22.65	4.85	<0.001
Rosenberg self-esteem scale	50	32.38	5.07	20	33.40	4.51	30	31.70	5.38	0.250
Subjective Happiness Scale	59	4.39	0.95	21	4.84	0.70	38	4.15	0.99	0.018
Satisfaction with Life Scale	39	18.10	4.33	16	20.00	3.22	23	16.78	4.58	0.037
Cognitive–Behavioral Avoidance Scale (CBAS)	59	2.54	0.77	21	2.85	0.91	38	2.36	0.63	0.034
Occupational Balance Questionnaire	59	50.96	15.36	21	59.09	14.72	38	46.47	13.95	0.002
Duke-UNC-11 Functional Social Support Questionnaire	59	39.35	10.07	21	42.85	8.29	38	37.42	10.54	0.046
Family Apgar scale	59	11.25	3.72	21	12.52	3.35	38	10.55	3.76	0.050
International Fitness Scale—general fitness	59	3.62	1.03	21	4.04	0.92	38	3.39	1.02	0.021
International Fitness Scale—cardiorespiratory fitness	59	3.91	0.87	21	4.19	0.92	38	3.76	0.81	0.057
International Fitness Scale—muscular strength	59	3.50	1.00	21	3.76	1.04	38	3.36	0.97	0.128
International Fitness Scale—speed–agility	59	3.57	0.96	21	3.85	1.01	38	3.42	0.91	0.109
International Fitness Scale—flexibility	59	3.49	1.11	21	3.66	1.19	38	3.39	1.07	0.313

**Table 3:** Association between HRQol and physical fitness perception, occupational balance, and self-esteem.

	B	B (95% CI)	β Standardized	T Statistic	*p*-Value	R^2^
**Model 1**						0.459
Constant	0.224	0.002 to 0.446		2.046	0.048	
International Fitness Scale—general fitness	0.162	0.105 to 0.219	0.688	5.764	0.000	
**Model 2**						0.596
Constant	−0.015	−0.247 to 0.218		−0.129	0.898	
International Fitness Scale—general fitness	0.137	0.085 to 0.188	0.580	5.416	0.000	
Occupational Balance Questionnaire	0.006	0.003 to 0.009	0.395	3.684	0.001	
**Model 3**						0.649
Constant	−0.289	−0.598 to 0.020		−1.897	0.066	
International Fitness Scale—general fitness	0.126	0.077 to 0.174	0.534	5.261	0.000	
Occupational Balance Questionnaire	0.006	0.003 to 0.009	0.375	3.742	0.001	
Rosenberg self-esteem scale	0.010	0.002 to 0.018	0.249	2.525	0.016	

## Data Availability

Data are contained within the article.

## References

[1] Nichols E., Steinmetz J.D., Vollset S.E., Fukutaki K., Chalek J., Abd-Allah F., Liu X. (2022). Estimation of the global prevalence of dementia in 2019 and forecasted prevalence in 2050: An analysis for the Global Burden of Disease Study 2019. Lancet Public Health.

[2] Gaugler J., James B., Johnson T., Reimer J., Solis M., Weuve J., Buckley R.F., Hohman T.J. (2022). 2022 Alzheimer’s disease facts and figures. Alzheimer’s Dement..

[3] Vitaliano P.P. (2010). An ironic tragedy: Are spouses of persons with dementia at higher risk for dementia than spouses of persons without dementia?. J. Am. Geriatr. Soc..

[4] Richardson T.J., Lee S.J., Berg-Weger M., Grossberg G.T. (2013). Caregiver health: Health of caregivers of Alzheimer’s and other dementia patients. Curr. Psychiatry Rep..

[5] Washington K.T., Meadows S.E., Elliott S.G., Koopman R.J. (2011). Information needs of informal caregivers of older adults with chronic health conditions. Patient Educ. Couns..

[6] Takai M., Takahashi M., Iwamitsu Y., Oishi S., Miyaoka H. (2011). Subjective experiences of family caregivers of patients with dementia as predictive factors of quality of life. Psychogeriatrics.

[7] Zarit S.H., Lee J.E., Barrineau M.J., Whitlatch C.J., Femia E.E. (2013). Fidelity and acceptability of an adaptive intervention for caregivers: An exploratory study. Aging Ment. Health.

[8] Yu H., Wang X., He R., Liang R., Zhou L. (2015). Measuring the Caregiver Burden of Caring for Community-Residing People with Alzheimer’s Disease. PLoS ONE.

[9] Kuo L.M., Huang H.L., Hsu W.C., Shyu Y.I. (2014). Health-related quality of life and self-efficacy of managing behavior problems for family caregivers of vascular dementia and Alzheimer’s disease patients. Dement. Geriatr. Cogn. Disord..

[10] Azzazy S., Riddle M. (2019). End of life care in inpatient psychiatry: A case study on end-stage alzheimer’s disease. Am. J. Geriatr. Psychiatry.

[11] Chhugani K.J., Metri K., Babu N., Nagendra H.R. (2018). Effects of Integrated Yoga Intervention on Psychopathologies and Sleep Quality Among Professional Caregivers of Older Adults with Alzheimer’s Disease: A Controlled Pilot Study. Adv. Mind-Body Med..

[12] Anderson J.G., Hundt E., Rose K.M. (2019). Nonpharmacological Strategies Used By Family Caregivers of Persons with Alzheimer’s Disease and Related Dementias as Presented in Blogs. J. Gerontol. Nurs..

[13] Martínez Marcos M., De la Cuesta Benjumea C. (2016). The experience of women care cargivers with chronic conditions of dependent relatives. Aten. Primaria.

[14] Argimon J.M., Limon E., Vila J., Cabezas C. (2004). Health-related quality of life in carers of patients with dementia. Fam. Pract..

[15] Prieto J.P. (2012). Efecto Sobre la Calidad de vida Relacionada con la Salud y la Condición Física de un Programa de Atención Domiciliaria Basado en Ejercicio Físico en Cuidadores de Personas con Demencia. Doctoral Dissertation.

[16] Tornal J.M.P., Martínez A.D. (2015). Influencia de las Emociones Positivas en la Salud de los Cuidadores de Enfermos de Alzheimer. Doctoral Dissertation.

[17] León-Salas B., Olazarán J., Muñiz R., González-Salvador M.T., Martínez-Martín P. (2011). Caregivers’ estimation of patients’ quality of life (QoL) in Alzheimer’s disease (AD): An approach using the ADRQL. Arch. Gerontol. Geriatr..

[18] Wister A.V., Coatta K.L., Schuurman N., Lear S.A., Rosin M., MacKey D. (2016). A Lifecourse Model of Multimorbidity Resilience: Theoretical and Research Developments. Int. J. Aging Hum. Dev..

[19] Durocher E., Gibson B.E., Rappolt S. (2021). Justicia Ocupacional: Una revisión de conceptos. J. Occup. Sci..

[20] Nah S., Martire L.M., Zhaoyang R. (2022). Perceived Gratitude, Role Overload, and Mental Health Among Spousal Caregivers of Older Adults. J. Gerontology. Ser. B Psychol. Sci. Soc. Sci..

[21] Röschel A., Wagner C., Dür M. (2022). Associations between occupational balance, subjective health, and well-being of informal caregivers of older persons based on a cross-sectional study. BMC Geriatr..

[22] Herdman M., Gudex C., Lloyd A., Janssen M., Kind P., Parkin D., Bonsel G., Badia X. (2011). Development and preliminary testing of the new five-level version of EQ-5D (EQ-5D-5L). Qual. Life Res..

[23] Ravens-Sieberer U., Wille N., Badia X., Bonsel G., Burström K., Cavrini G., Devlin N., Egmar A.-C., Gusi N., Herdman M. (2010). Feasibility, reliability, and validity of the EQ-5D-Y: Results from a multinational study. Qual. Life Res..

[24] Hernandez G., Garin O., Pardo Y., Vilagut G., Pont À., Suárez M., Neira M., Rajmil L., Gorostiza I., Ramallo-Fariña Y. (2018). Validity of the EQ–5D–5L and reference norms for the Spanish population. Qual. Life Res..

[25] Merino-Soto C., Angulo-Ramos M. (2013). Validación en Chile de la escala de sobrecarga del cuidador de Zarit en sus versiones original y abreviada: Corrección. Rev. Médica Chile.

[26] Zarit S.H., Reever K.E., Bach-Peterson J. (1980). Relatives of the impaired elderly: Correlates of feelings of burden. Gerontology.

[27] Higginson I.J., Gao W., Jackson D., Murray J., Harding R. (2010). Short-form Zarit Caregiver Burden Interviews were valid in advanced conditions. J. Clin. Epidemiol..

[28] Vázquez-Morejón Jiménez R., Jiménez García-Bóveda R., Vázquez Morejón A.J. (2004). Escala de autoestima de Rosenberg: Fiabilidad y validez en población clínica española. Apunt. Psicol..

[29] Mayordomo T., Gutierrez M., Sales A. (2020). Adapting and validating the Rosenberg Self-Esteem Scale for elderly Spanish population. Int. Psychogeriatr..

[30] Lyubomirsky S., Lepper H.S. (1999). A measure of subjective happiness: Preliminary reliability and construct validation. Soc. Indic. Res..

[31] Extremera N., Fernández-Berrocal P. (2014). The Subjective Happiness Scale: Translation and preliminary psychometric evaluation of a Spanish version. Soc. Indic. Res..

[32] Quezada L., Landero R., González M.T. (2016). A validity and reliability study of the Subjective Happiness Scale in Mexico. J. Happiness Well-Being.

[33] Feliu-Soler A., de Diego-Adeliño J., Luciano J.V., Iraurgi I., Alemany C., Puigdemont D., Pérez V., Portella M.J., Trujols J. (2021). Unhappy while depressed: Examining the dimensionality, reliability and validity of the subjective happiness scale in a spanish sample of patients with depressive disorders. Int. J. Environ. Res. Public Health.

[34] Diener E., Emmons R.A., Larsen R.J., Griffin S. (1985). The satisfaction with life scale. J. Personal. Assess..

[35] Vázquez C., Duque A., Hervás G. (2013). Satisfaction with life scale in a representative sample of Spanish adults: Validation and normative data. Span. J. Psychol..

[36] Bagherzadeh M., Loewe N., Mouawad R.G., Batista-Foguet J.M., Araya-Castillo L., Thieme C. (2018). Spanish version of the Satisfaction with Life Scale: Validation and factorial invariance analysis in Chile. Span. J. Psychol..

[37] Ruiz F.J., Suárez-Falcón J.C., Flórez C.L., Odriozola-González P., Tovar D., López-González S., Baeza-Martín R. (2019). Validity of the Satisfaction with Life Scale in Colombia and factorial equivalence with Spanish data. Rev. Latinoam. Psicol..

[38] Hernández-Guzmán L., Dobson K.S., Caso-Niebla J., González-Montesinos M., Epp A., Arratíbel-Siles M.L., Wierzbicka-Szymczak E. (2009). La versión en español de la Escala Cognitivo-Conductual de Evitación (CBAS). Rev. Latinoam. Psicol..

[39] Barajas S., Garra L., Ros L. (2017). Avoidance in anxiety and depression: Adaptation of the cognitive-behavioral avoidance scale in a Spanish sample. Span. J. Psychol..

[40] Gómez P.P. (2017). Equilibrio Ocupacional en Estudiantes de Terapia Ocupacional. Doctoral Dissertation.

[41] Peral-Gómez P., Espinosa-Sempere C., Navarrete-Muñoz E.M., Hurtado-Pomares M., Juárez-Leal I., Valera-Gran D., Sánchez-Pérez A. (2022). The Spanish version of Occupational Balance Questionnaire: Psychometric properties and normative data in a representative sample of adults. Ann. Med..

[42] Bellón Saameño J.A., Delgado Sánchez A., Luna del Castillo J.D., Lardelli Claret P. (1996). Validity and reliability of the Duke-UNC-11 questionnaire of functional social support. Aten. Primaria.

[43] Cuéllar-Flores I., Dresch V. (2012). Validación del cuestionario de Apoyo Social Funcional Duke-UNK-11 en personas cuidadoras. Rev. Iberoam. Diagnóstico Y Evaluación-E Avaliação Psicológica.

[44] Bellón Saameño J.A., Delgado Sánchez A., Luna del Castillo J.D., Lardelli Claret P. (1996). Validity and reliability of the family Apgar family function test. Aten. Primaria.

[45] Grimaldos J.A.J. (2023). Confiabilidad y validez del APGAR familiar como instrumento de evaluación de la. Urbano.

[46] Valencia-Vargas A., López-Palacio G.J., Cardona-Arango D., Segura-Cardona Á.M., Segura-Cardona A., Muñoz-Rodríguez D.I., Rojas-Gualdrón D.F. (2021). Análisis Rasch de la escala APGAR-familiar en adultos mayores de Colombia. Hacia Promoc. Salud.

[47] Mayorga-Munoz C., Gallardo-Peralta L., Galvez-Nieto J.L. (2019). Psychometric properties of APGAR-family scale in a multiethnic sample of Chilean older people. Rev. Médica Chile.

[48] Ortega F.B., Ruiz J.R., Espana-Romero V., Vicente-Rodriguez G., Martínez-Gómez D., Manios Y., Béghin L., Molnar D., Widhalm K., Moreno L.A. (2011). The International Fitness Scale (IFIS): Usefulness of self-reported fitness in youth. Int. J. Eat. Disord..

[49] Merellano-Navarro E., Collado-Mateo D., García-Rubio J., Gusi N., Olivares P.R. (2017). Validity of the International Fitness Scale “IFIS” in older adults. Exp. Gerontol..

[50] Ortega F., Sánchez-López M., Solera-Martínez M., Fernandez-Sanchez A., Sjöström M., Martinez-Vizcaino V. (2013). Self-reported and measured cardiorespiratory fitness similarly predict cardiovascular disease risk in young adults. Scand. J. Med. Sci. Sports.

[51] Liao X., Huang Y., Zhang Z., Zhong S., Xie G., Wang L., Xiao H. (2020). Factors associated with health-related quality of life among family caregivers of people with Alzheimer’s disease. Psychogeriatrics.

[52] van Hezik-Wester V.J., Handels R.L.H., Wolfs C.A.G., Kanters T.A. (2023). Caregiver Burden and Quality of Life Across Alzheimer’s Disease Severity Stages. Alzheimer Dis. Assoc. Disord..

[53] Gruffydd E., Randle J. (2006). Alzheimer’s disease and the psychosocial burden for caregivers. Community Pract..

[54] American Occupational Therapy Association (2020). Occupational Therapy Practice Framework: Domain et Process.

[55] Toribio-Díaz M., Medrano-Martínez V., Moltó-Jordá J., Beltrán-Blasco I.J.N. (2013). Red de cuidadores informales de los pacientes con demencia en la provincia de Alicante, descripción de sus características. Neurología.

[56] Tarlow B.J., Wisniewski S.R., Belle S.H., Rubert M., Ory M.G., Gallagher-Thompson D. (2004). Positive aspects of caregiving: Contributions of the REACH project to the development of new measures for Alzheimer’s caregiving. Res. Aging.

[57] Pereira M.G., Abreu A.R., Rego D., Ferreira G., Lima S. (2021). Contributors and Moderators of Quality of Life in Caregivers of Alzheimer’s Disease Patients. Exp. Aging Res..

[58] Kim H., Lee Y.W. (2014). P3-370: Predictors of quality of life among primary caregivers of dementia elderly. Alzheimer’s Dement..

[59] Bergvall N., Brinck P., Eek D., Gustavsson A., Wimo A., Winblad B., Jönsson L. (2011). Relative importance of patient disease indicators on informal care and caregiver burden in Alzheimer’s disease. Int. Psychogeriatr..

[60] Pudelewicz A., Talarska D., Bączyk G. (2019). Burden of caregivers of patients with Alzheimer’s disease. Scand. J. Caring Sci..

[61] Hernández-Padilla J.M., Ruiz-Fernández M.D., Granero-Molina J., Ortíz-Amo R., López Rodríguez M.M., Fernández-Sola C. (2021). Perceived health, caregiver overload and perceived social support in family caregivers of patients with Alzheimer’s: Gender differences. Health Soc. Care Community.

[62] Rosende-Roca M., Cañabate P., Moreno M., Preckler S., Seguer S., Esteban E., Tartari J.P., Vargas L., Narvaiza L., Pytel V. (2022). Sex, Neuropsychiatric Profiles, and Caregiver Burden in Alzheimer’s Disease Dementia: A Latent Class Analysis. J. Alzheimer’s Dis. JAD.

[63] Cheng S.-T., Au A., Losada A., Thompson L.W., Gallagher-Thompson D. (2019). Psychological interventions for dementia caregivers: What we have achieved, what we have learned. Curr. Psychiatry Rep..

[64] Hovbrandt P., Håkansson C., Albin M., Carlsson G., Nilsson K. (2019). Prerequisites and driving forces behind an extended working life among older workers. Scand. J. Occup. Ther..

[65] Rolland Y., Czerwinski S., Abellan Van Kan G., Morley J.E., Cesari M., Onder G., Woo J., Baumgartner R., Pillard F., Boirie Y. (2008). Sarcopenia: Its assessment, etiology, pathogenesis, consequences and future perspectives. J. Nutr. Health Aging.

[66] Andreakou M.I., Papadopoulos A.A., Panagiotakos D.B., Niakas D. (2016). Assessment of Health-Related Quality of Life for Caregivers of Alzheimer’s Disease Patients. Int. J. Alzheimer’s Dis..

